# Broad external validation of a multivariable risk prediction model for gastrointestinal malignancy in iron deficiency anaemia

**DOI:** 10.1186/s41512-021-00112-8

**Published:** 2021-12-15

**Authors:** Orouba Almilaji, Gwilym Webb, Alec Maynard, Thomas P. Chapman, Brian S. F. Shine, Antony J. Ellis, John Hebden, Sharon Docherty, Elizabeth J. Williams, Jonathon Snook

**Affiliations:** 1 Gastroenterology Unit, Poole Hospital, University Hospitals Dorset NHS Foundation Trust, Poole, UK; 2 Department of Medical Science and Public Health, Bournemouth University, Bournemouth, UK; 3 Cambridge Liver Unit, Addenbrooke’s Hospital, Cambridge University Hospitals NHS Foundation Trust, Cambridge, UK; 4 Weston Park Cancer Centre, Sheffield Teaching Hospitals NHS Foundation Trust, Sheffield, UK; 5 Department of Gastroenterology, Worthing Hospital, University Hospitals Sussex NHS Foundation Trust, Worthing, UK; 6 Nuffield Department of Clinical Laboratory Sciences, University of Oxford, Oxford, UK; 7 Translational Gastroenterology Unit, NIHR Oxford Biomedical Research Centre, University of Oxford, Oxford, UK; 8 Northern General Hospital, Sheffield Teaching Hospitals NHS Foundation Trust, Sheffield, UK

**Keywords:** Iron deficiency anaemia, Gastrointestinal cancer, IDIOM app, External validation, Temporal validation, TRIPOD

## Abstract

**Background:**

Using two large datasets from Dorset, we previously reported an internally validated multivariable risk model for predicting the risk of GI malignancy in IDA—the IDIOM score. The aim of this retrospective observational study was to validate the IDIOM model using two independent external datasets.

**Methods:**

The external validation datasets were collected, in a secondary care setting, by different investigators from cohorts in Oxford and Sheffield derived under different circumstances, comprising 1117 and 474 patients with confirmed IDA respectively. The data were anonymised prior to analysis. The predictive performance of the original model was evaluated by estimating measures of calibration, discrimination and clinical utility using the validation datasets.

**Results:**

The discrimination of the original model using the external validation data was 70% (95% CI 65, 75) for the Oxford dataset and 70% (95% CI 61, 79) for the Sheffield dataset. The analysis of mean, weak, flexible and across the risk groups’ calibration showed no tendency for under or over-estimated risks in the combined validation data. Decision curve analysis demonstrated the clinical value of the IDIOM model with a net benefit that is higher than ‘investigate all’ and ‘investigate no-one’ strategies up to a threshold of 18% in the combined validation data, using a risk cut-off of around 1.2% to categorise patients into the very low risk group showed that none of the patients stratified in this risk group proved to have GI cancer on investigation in the validation datasets.

**Conclusion:**

This external validation exercise has shown promising results for the IDIOM model in predicting the risk of underlying GI malignancy in independent IDA datasets collected in different clinical settings.

**Supplementary Information:**

The online version contains supplementary material available at 10.1186/s41512-021-00112-8.

## Background

The strong association between iron deficiency anaemia (IDA) and gastrointestinal (GI) cancer is well recognised [1–5]. As a result, IDA in at-risk groups is an accepted indication for fast-track referral to secondary care for further investigation in the UK [6]. The problem with this approach is that IDA is common, but the prevalence of malignancy is only 8-10% [7]—meaning a large workload for a relatively small yield.

With the aim of risk stratification, we have previously built and internally validated a binary multivariable logistic model to predict the risk of GI cancer in patients with confirmed IDA, based on four simple variables: age, sex, haemoglobin concentration (Hb) and mean cell volume (MCV)—the IDIOM model (iron deficiency as an indicator of malignancy) [4]. Identifying subgroups of IDA patients who are at increased/reduced risk of GI cancer might lead to (a) accelerating the investigation of those at high predicted risk, with potential prognostic implications, and (b) helping those at low predicted risk to avoid unnecessary invasive procedures.

The clinical data used to develop the original model was collected for adult patients with IDA (*n* = 1879) referred to the IDA clinic in Poole hospital during the period 2004-2016 inclusive. The criteria for inclusion were iron deficiency confirmed by standard laboratory criteria, and subsequent investigation of the upper and lower GI tract. Due to informed patient preference, concurrent illness or major co-morbidity, about 10% of IDA patients usually fail to undergo GI investigation for IDA [3, 8].

Developing the original model was carried out before receiving an internal dataset (*n* = 511) from the later period 2017-2018. Using this dataset, temporal validation of the fitted model showed excellent promise of generalisability. The area under the receiver operating characteristic (ROC) curve (AUC) was estimated at 81% (95% CI 74, 86). The prevalence of malignancy was 8.4% in the temporal dataset. The average estimated risk of 7.6% indicated that the IDIOM model has no tendency to underestimate or overestimate risk. The calibration intercept and slope were 0.1 (95% CI −0.1, 0.4) and 1.1 (95% CI 0.8, 1.5) respectively, suggesting that risk estimates were not systematically too moderate or extreme.

However, the data used to validate the model were collected by the same centre (IDA clinic in Poole hospital), for the same population (Dorset), using the same predictors and outcome definitions and measurements. Confirmed IDA was defined using the same blood laboratory marker cut-offs in the training and internal validation datasets, but these cut-offs are relevant only to the local laboratory and may vary between laboratories.

So to apply the model with confidence to different populations it must be tested, and amended in case of poor performance, using data collected by other investigators in other geographic areas and preferably using locality-specific definitions for the predictors. This retrospective cohort study aims to address the transportability of IDIOM score model by broadly validating it using two independent external datasets.

## Methods

After temporally validating the model in 2020, the training and internal datasets were merged to form the Dorset dataset. This was used to fit the full IDIOM model [9]. The multiple binary logistic regression of this full model was constructed according to the formula:

$$ \log \left\{\frac{\mathbb{P}\left( GI\  Maligancy= postive\right)}{\mathbb{P}\left( GI\  Maligancy= negative\right)}\right\}=-1.84+0.94\  sex+\kern0.5em 0.06\  age-0.03\  MCV-0.03\  Hb $$

The full IDIOM model was almost identical to the original model using only the training dataset [4]. Statistical assessment of the validity and goodness of fit of the logistic regression model (smoothed scatter plot, deviance and residual test, Cook’s distance and standardised residual errors, variance inflation factor, Akaike information criterion, analysis of variance *χ*^2^ test, pseudo *R*^2^) was satisfactory.

Before importing the coefficients of the full IDIOM model to predict the risk of GI cancer in the validation data, least absolute shrinkage and selection operator (Lasso) was applied to regulate the model. A comparison of different regularisation method effects on the model coefficients is shown in Additional file, Table S1. The model coefficients after applying these methods were very close; however, Lasso method was selected because it is the method that shrunk the coefficients the most.

The final updated multiple binary logistic regression of the full IDIOM model regulated using Lasso method and validated in this study was constructed according to the formula:

$$ \log \left\{\frac{\mathbb{P}\left( GI\  Maligancy= postive\right)}{\mathbb{P}\left( GI\  Maligancy= nega\mathrm{t} ive\right)}\right\}=-1.84+0.89\  sex+\kern0.5em 0.05\  age-0.03\  MCV-0.06\  Hb $$

The ORs (95% CI, *p* value) for the four predictive variables were as follows:

- Sex: 2.44 for men (1.88 to 3.49, *p* < 0.0001)
- Age: 1.05 per year (1.04 to 1.08, *p* < 0.0001)
- MCV: 1.03 for each fl reduction (1.01 to 1.05, *p* < 0.01)
- Hb: 1.03 for each g/l reduction (1.02 to 1.04, *p* < 0.0001)

The quartiles of positive predictive values (PPV) were updated based on the penalised model (Table 1). The first PPV quarter was divided into two halves, in which the lower half corresponds to negative predictive values (NPV) equal to 100% only. The highest predicted risks in each PPV quarter (and the lower half of the first quarter) were used as cut-offs to create the risk groups. The updated cut-offs to create the risk groups were 1.18%, 2.16%, 4.24% and 7.97%.

**Table 1:** Risk groups cut-offs after regulating IDIOM model based on the quartiles of PPV

PPV quarters	PPV values range %	Corresponding predicted risk cut-offs %	Risk group
Lower half of the 1st quarter of PPV^a^	[8.4-9.4]	≤ 1.18	Very low risk
Upper half of the 1st quarter of PPV	]9.4-10.8]	]1.18-2.16]	Low risk
2nd quarter of PPV	]10.8-14.7]	]2.16-4.24]	Moderate risk
3rd quarter of PPV	]14.7-19.6]	]4.24-7.97]	High risk
4th quarter of PPV	> 19.6	> 7.97	Very high risk

^a^The risk group at which PPV values are in the lower quarter, and NPV = 100

PPV is the number of positive cases that were correctly classified divided by the total number of positive cases predicted. NPV is the number of negative cases that were correctly classified divided by the total number of negative cases predicted

The highest Gmean (geometric mean of sensitivity and specificity) value in the Dorset dataset was updated using the penalised IDIOM model and found to be around 70%.

### Source of data

Independent datasets were collected by investigators in Oxford and Sheffield and included all subjects who met the inclusion criteria within the collection time frame. The Oxford dataset was collected for the period 2016-2019 and comprised 1147 subjects with IDA referred for fast-track investigation. The Sheffield dataset was collected for the period 2013-2018 and compromised 477 subjects with IDA referred to a dedicated IDA Clinic.

### Patients

For all datasets, the subjects were adults referred to secondary care who went on to be investigated for IDA. The decision to refer was generally made in primary care by the GP who requested the blood test revealing IDA, usually following a discussion with the patient concerned about the significance and potential implications of the result.

Confirmation of IDA depended on local practice but was broadly accepted as: (a) Transferrin saturation (T.sat) < 15% and/or serum ferritin less than the lower laboratory limit of normal at the time for the Dorset dataset, (b) T.sat < 16% and/or serum ferritin < 10 μg/l (women) or < 20 μg/l (men) for the Oxford dataset, (c) serum ferritin < 31 μg/l (both sexes) for the Sheffield dataset. The diagnosis of iron deficiency was confirmed in all subject in each of the datasets by the finding of an abnormally low serum ferritin and/or transferrin saturation. All subjects underwent standard first-line GI investigation for IDA, comprising exclusion of coeliac disease, OGD and an adequate colonic examination—either by CT colonography or colonoscopy.

### Outcome and predictors

As for the IDIOM model, the outcome was the presence/absence of cancer of the upper or lower GI tract. The predictors were the recoded values of age at presentation (years), sex (male/female), Hb (g/l) and MCV (fl) measured from the same blood sample taken prior to iron replacement therapy. The decision regarding the presence or absence of GI malignancy was made by clinician with responsibility for the case after GI investigations were complete.

### Sample size

Being a retrospective analysis of secondary data meant that there was no control of the size of the external validation datasets. The number of outcome events in the Oxford and Sheffield datasets was 86, 36 respectively. Following the simulation-based sample size calculations for external validation of clinical prediction models [10], the anticipated precisions of performance measures were estimated based on the available number of outcome events in each external validation dataset, and on them both combined. Further details about sample size considerations are included in Additional file (sample size, Fig. S1, Fig. S2).

### Development vs. validation(s)

Differences in the quoted normal ranges for Hb, MCV, T.sat and serum ferritin were to be expected between the laboratories in Dorset, Oxford and Sheffield as these references are relevant only to the local laboratory. However, the differences for all the variables (as shown in Additional file, Table S2) were marginal.

### Missing data

There was no missing data in the external validation datasets for the results of IDA investigations, Hb, MCV, sex and age.

### Statistical analysis

Before starting the analysis, external validation datasets were prepared by taking out duplicates and applying inclusion (confirmed IDA patients who underwent standard first-line GI investigation for IDA) and exclusion criteria (all IDA patients diagnosed with other malignancies, e.g. ovarian cancers, renal cancers, GIST, neuroendocrine tumours were excluded). The updated cut-offs used to create the risk groups in Dorset (Table 1) were imported to create risk groups in Oxford, Sheffield and the combined validation datasets and then the predictive performance of the IDIOM model was evaluated using the validation datasets by estimating the following measures:

#### Discrimination

Discrimination refers to the ability of the model to distinguish correctly between the presence and absence of GI cancer in the validation datasets. Discrimination of the IDIOM model was assessed by examining the values of C-statistic for these datasets. For a binary outcome, the C-statistic is equivalent to the area under the receiver operating characteristic (ROC) curve (AUC). The highest Gmean values in the Dorset, Oxford, Sheffield and the combined validation datasets were compared visually by adding them to the ROC curve graph.

#### Calibration

Calibration quantifies how close estimated risks are to observed ones in the validation datasets. Assessment of IDIOM calibration was carried out following published methodology [11], employing mean calibration (or calibration-in-the-large), weak calibration (calibration intercept and calibration slope) and moderate calibration (flexible calibration curve based on Loess functions).

To check calibration across the risk groups, we split the combined validation dataset based on descending order of probabilities into fifths (5 groups) using the defined cut-offs (in Table 1). Then, the calibration between observed and predicted risks across the risk groups was assessed visually using a calibration plot. As per the sample size considerations (additional file), the two external datasets were combined to assess the calibration.

#### Net benefit

The net benefit (NB) expresses the relative value of benefits and harms associated with using the model. Benefits reflect the diagnosis of a GI cancer by investigation, whilst harms include the risks and cost of carrying out an unnecessary invasive investigation.

Since the current standard of care is to offer investigation to all patients with IDA at risk of malignancy, a major potential use of the IDIOM risk model would be to identify those at very low risk who may not warrant investigation. Decision curves can be used as a tool for assessing the performance of risk prediction models [12]. Decision curves were used to assess the clinical value of the model by ensuring that it had a higher NB than simple strategies such as “investigate all” or “investigate no-one” across a plausible range of risk thresholds. Clinical impact curves, which are alternative plots for the outputs of decision curves, were used to compare the estimated number of patients who would be classified as low risk, and the number of patients classified low risk without the outcome of interest (true negative) at each threshold.

Subjects diagnosed with GI cancer (cases) have expected benefit *B* > 0 from the investigation, where *B* accounts for the totality of good and bad effects. Likewise, subjects who do not have GI cancer (controls) have a cost (or burden) of the investigation, *C* > 0 [13]. Given benefit (*B*) and cost (*C*), the optimal risk threshold (*R*) for determining investigation is:

$$ R=\frac{C}{C+B} $$

When the policy is ‘investigate all’, all controls experience the cost of investigation. The advantage of an opt-out policy to the patient population accrues from controls whose estimated risks are below *R*, as such patients avoid the cost [13]. Expressing NB in terms of avoided unnecessary investigations is recommended if the reference strategy is ‘investigate all’, and so NB is expressed in terms of true negatives rather than true positives [14]. Given that *ρ* is the proportion of cases, the standardised net benefit—which is easier to be interpreted than net benefit—can be calculated by dividing the net benefit by (1 − *ρ*) as can be shown from the equation [13]:

$$ sNB={TNR}_R-\kern0.5em \frac{\rho }{\left(1-\rho \right)}\frac{1-R}{R}\ {FNR}_R $$

In which TNR is the specificity at a given risk threshold *R*, and FNR is the miss rate at the same threshold. At ‘investigate no-one’; TNR = FNR ≡ 1, and at ‘investigate all’; TNR = FNR ≡ 0. In an ‘investigate all’ standard of care, the standardised net benefit can be viewed as the TNR appropriately discounted by the FNR [13]. With an ‘investigate all’ standard of care, it is difficult for any model to perform better than a strategy of ‘investigate no-one’ when the prevalence is low, and so for this analysis, we combined both external validation datasets into one and compared that to the Dorset dataset.

The TRIPOD (transparent reporting of a multivariable prediction model for individual prognosis or diagnosis) initiative was followed to report this study [15]. R (version 3.6.1) and RStudio (version 1.2.5001) were used to run the statistical analysis and to produce the graphs.

## Results

### Patients

After tidying the databases and applying the exclusion criteria, 1117 cases were available for detailed analysis from the Oxford dataset and 474 from the Sheffield dataset. There were differences between the datasets, as shown in Table 2. As expected, the Oxford dataset had a lower median Hb in particular, as subjects presented exclusively through the fast-track pathway.

**Table 2:** Descriptive statistics for the three datasets

Dataset		Dorset	Oxford	Sheffield
Dataset size	***N***	2390	1117	474
**GI cancer**	**Positive—*n*** **(%)**	200 (8.4%)	86 (7.7%)	36 (7.6%)
**Sex**	**Male—*n*** **(%)**	862 (36%)	446 (40%)	227 (48%)
**Age (years)**	**Median (min, max)**	71 (16, 96)	74 (22, 97)	69 (18, 93)
**Hb (g/l)**	**Median (min, max)**	104 (32, 159)	91 (29, 129)	104 (54, 152)
**MCV (fl)**	**Median (min, max)**	80 (53, 112)	81 (55, 125)	80 (32, 104)

A density plot of continuous variables according to the presence/absence of GI cancer in each dataset using the IDIOM model is illustrated in Additional file, Fig. S3 whilst the probability distributions for each dataset are shown in Additional file, Fig. S4.

### Model performance

#### Discrimination

The discrimination of the IDIOM model was AUC, 77% (95% CI 74, 80) for the Dorset dataset; AUC, 70% (95% CI 65, 75) for the Oxford dataset; AUC, 70% (95% CI 61, 79) for the Sheffield dataset and AUC, 69% (95% CI 65, 74) for the combined validation dataset. As predicted by the sample size calculations, due to the small sample size of the Sheffield dataset, the width of the CI for the discrimination was the largest. And for the combined validation data, was less than 10%.

Using the risk groups cut-offs in Table 1, analysis showed the following:

- Cut-off 1 (≤ 1.18%) stratified about 11% of the Dorset dataset and 3% of both external cohorts (Oxford, 2%; Sheffield, 5%) into the very low risk group.
- Cut-off 2 (1.18-2.16%) stratified about 14% of the Dorset dataset and 8% of both external cohorts (Oxford, 7%; Sheffield, 12%) into the low risk group.
- Cut-off 3 (2.16-4.24%) stratified about 26% of the Dorset dataset and 25% of both external cohorts (Oxford, 22%; Sheffield, 31%) into the moderate risk group.
- Cut-off 4 (4.24-7.97%) stratified about 24% of the Dorset dataset and 31% of both external cohorts (Oxford, 30%; Sheffield, 33%) into the high risk group.
- Cut-off 5 (> 7.97%) stratified about 25% of the Dorset dataset and 33% of both external cohorts (Oxford, 39%; Sheffield, 19%) into the very high risk group.

The proportion of patients who fell into the higher-risk groups from the Oxford dataset was large (69%). This was expected because the patients in the Oxford dataset had lower Hb values. None of the patients stratified in the very low risk group from the validation datasets (Oxford, 2%; Sheffield, 5%) proved to have GI cancer on investigation as NPV remains 100%.

**Fig. 1:**
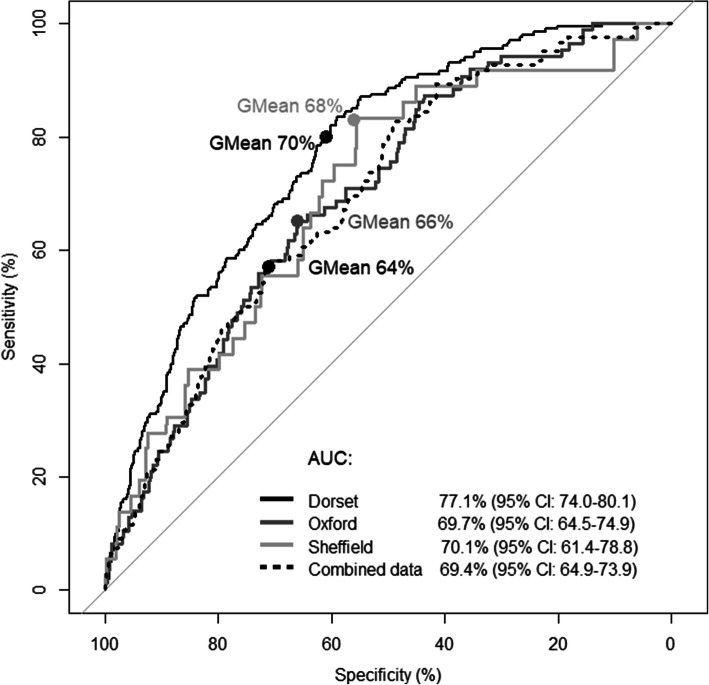
Receiver operating characteristic curve shows the sensitivity on *y*-axis, and specificity on *x*-axis for the Dorset (black), Oxford (dark grey), Sheffield (grey) and combined validation (dotted black) datasets with the highest GMean value in each dataset. AUC, area under curve; GMean, geometric mean of sensitivity and specificity

The ROC curve (Fig. 1) showed that the highest Gmean values in the validation datasets were close (70% in the Dorset dataset, 66% in the Oxford dataset, 68% in the Sheffield dataset and 64% in the combined dataset).

#### Calibration

##### Risk groups calibration

Assessing the calibration visually across the five risk groups in the combined validation data suggested (Additional file, Fig. S5) that the observed and predicted risks across the five risk groups were overall similar.

##### Mean calibration (calibration-in-the-large)

The prevalence of malignancy was 7.7% (86/1117) for the Oxford series, 7.6% (36/474) for the Sheffield series and 7.7% (122/1591) for the combined datasets. The average risks estimated by the IDIOM model were 8.5%, 5.5% and 7.6% respectively. Assessing the risk ratios, using the validation datasets separately, showed that there was an overestimation for the risks in the Oxford dataset (by 10%), and underestimation for the risk in the Sheffield dataset (by 28%). However, the analysis using the combined validation dataset showed no tendency for the model to under- or overestimate risk (by 1%).

##### Weak calibration (calibration intercept and calibration slope)

For the Oxford dataset, the calibration intercept and slope were −0.11 (95% CI −0.34, 0.12), and 0.87 (95% CI 0.59, 1.15) respectively. For the Sheffield dataset, the calibration intercept and slope were 0.35 (95% CI 0.01, 0.70) and 0.96 (95% CI 0.5, 1.42) respectively. For the combined Oxford and Sheffield datasets, the number of events was 122, the calibration intercept and slope were 0.01 (95% CI, −0.18, 0.20) and 0.84 (95% CI 0.60, 1.07) respectively. With zero as the target value for the intercept, the results for Oxford dataset and the combined data suggest no tendency for under- or overestimated risks. The calibration slopes were close to the target value of 1, suggesting that risk estimates for Oxford dataset and the combined data were not systematically too moderate or extreme in either dataset.

The confidence intervals for the calibration intercept all contain 0 apart from the small size Sheffield dataset. All confidence intervals were wide > 0.2; however, this result was consistent with what the sample size calculations predicted based on the existing relatively small number of outcome events.

##### Moderate calibration (flexible calibration curve)

**Fig. 2:**
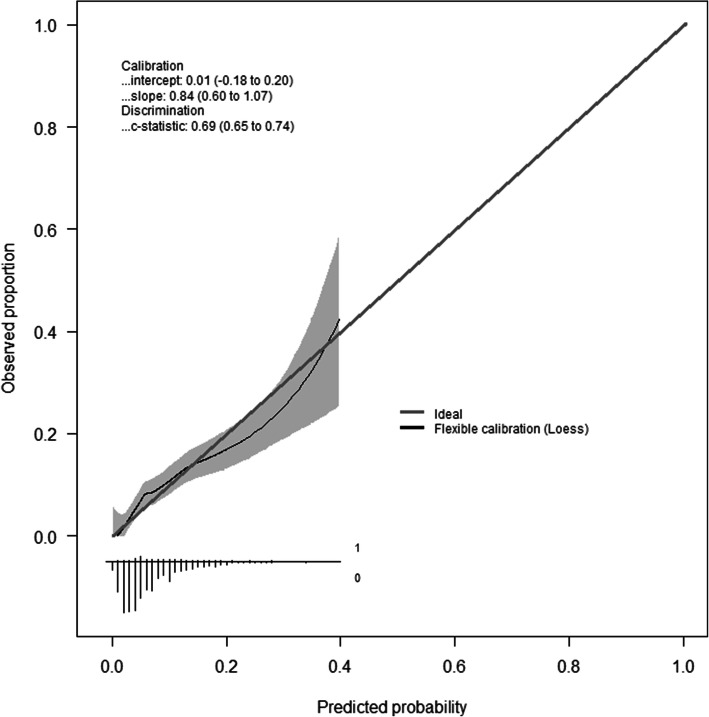
Flexible calibration curve for the combined external datasets, showing the relationship between the estimated risks (on the *x*-axis) and the observed proportion of events (on the *y*-axis)

The flexible calibration plot for the combined validation dataset (Fig. 2) showed that the model was well-calibrated for risks up to about 17%, but miscalibrated for a few of the higher risk patients. For example, in Fig. 2, a predicted risk of 30% corresponds to an observed risk of around 20%. However, about 92% of the combined cohort patients have predicted risks less than 17.5% and the model is well-calibrated in this region. Also, using any of these cut-off values above 17.5% would put these patients in the very high-risk group regardless of their predicted risks.

The calibration plot for the Oxford dataset (Additional file, Fig. S6) showed similar results to that in the combined validation dataset (in which 90% of the Oxford cohort patients have predicted risks less than 17.5%).

Furthermore, the flexible calibration plot for the Sheffield dataset (Additional file, Fig. S7) showed a strong deviation from the ideal line across the range of true risks above 20%. The miscalibration above 17.5% was consistent with the previous results in the Oxford and combined datasets. However, only 2% of the Sheffield cohort has predicted risks more than 17.5%.

#### Net benefit

Decision curve analysis suggested that the IDIOM model is of clinical value because it has the potential to add value—i.e., standardised NB is higher than ‘investigate no-one’ and ‘investigate all’ for a range of risk thresholds up to 27% in the Dorset dataset, up to 18% in the combined validation dataset (and up to 18% in the Oxford dataset, and to 18% in the Sheffield dataset as can be seen from Additional file, Figs. S8 and S9).

**Fig. 3:**
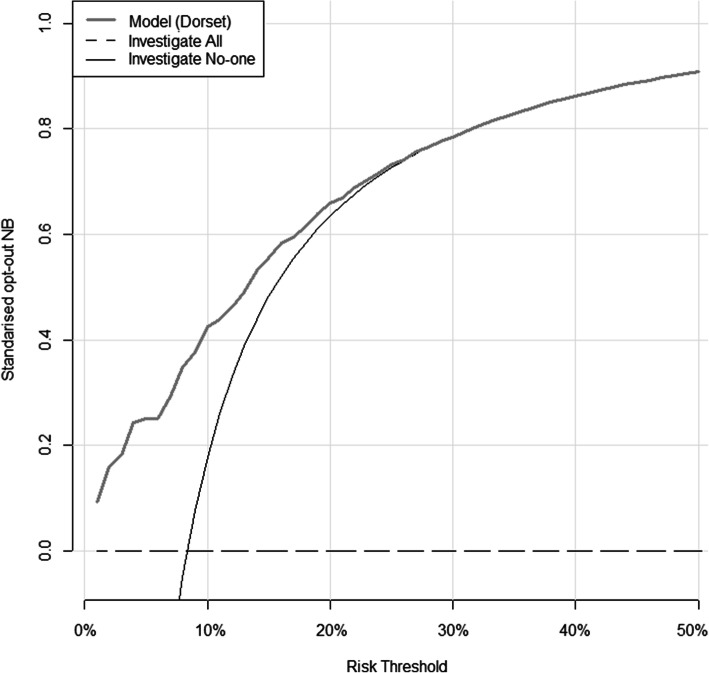
Decision curve analysis for GI investigation using Dorset data. Grey line: penalised IDIOM model. Black line: investigate no-one strategy. Dashed line: investigate all strategy. The vertical axis displays standardised net benefit. The horizontal axis shows the risk thresholds

**Fig. 4:**
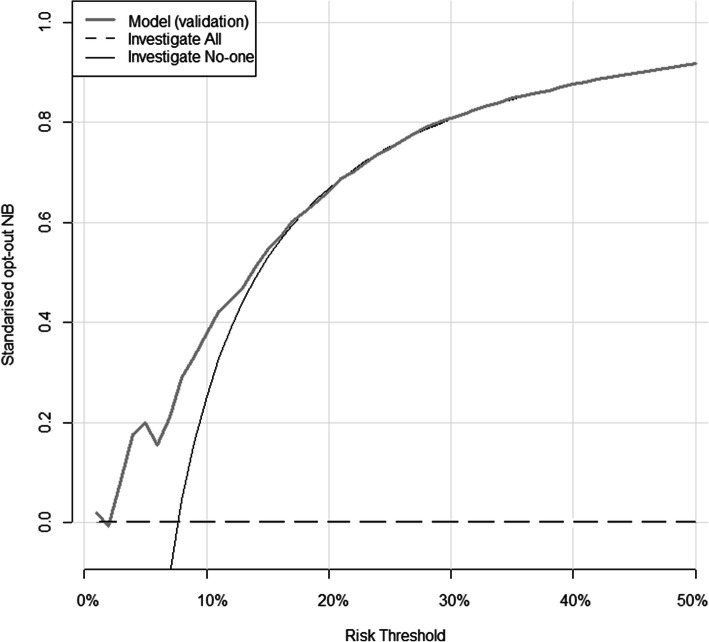
Decision curve analysis for GI investigation using the combined external datasets. Grey line: penalised IDIOM model. Black line: ‘investigate no-one’ strategy. Dashed line: ‘investigate all’ strategy. The vertical axis displays standardised net benefit. The horizontal axis shows the risk thresholds

So at a risk threshold of 10% for example, use of the IDIOM model would be the equivalent to a theoretical strategy that reduced the number of unnecessary investigations by about 43 per 100 in the Dorset dataset (Fig. 3), and 38 per 100 in the combined validation dataset (38 per 100 in the Oxford dataset, 37 per 100 in the Sheffield dataset) (Fig. 4).

The clinical impact curves (Additional file, Figs. S10 and S11) showed that at a risk threshold of 10%, around 825 of 1000 IDA patients in the Dorset dataset would be anticipated as low risk and about 780 of these as true negatives for GI cancer (cost/benefit: 1/9). At the same risk threshold, of 1000 IDA patients in the combined validation dataset, about 750 would be predicted as low risk and about 710 of them as true negatives for GI cancer (cost/benefit: 1/9).

Figures S12 and S13 (Additional file) showed that at the same risk threshold of 10%, around 690 of 1000 IDA patients in the Oxford dataset would be anticipated as low risk and about 650 of these as true negatives for GI cancer (cost/benefit: 1/9). At the same risk threshold, of 1000 IDA patients in the Sheffield validation dataset, about 870 would be predicted as low risk and about 840 of them as true negatives for GI cancer (cost/benefit: 1/9).

Regardless of the sample size, in every dataset, the net benefit analysis has shown consistently a clinical value for the IDIOM model in the validation datasets.

## Discussion

IDA is a problem commonly encountered in clinical practice, and the prevalence of underlying GI cancer is the primary justification for the urgent investigation of it [7, 16–21]. Bidirectional endoscopy, combining gastroscopy and colonoscopy in the same session, is generally accepted as the most efficient method of assessing the GI tract unless there are clear clinical clues as to the cause [20]. It does however carry a small but significant risk of complications, particularly in the elderly and those with major comorbidities, and it is therefore important to consider the risk-benefit ratio for the investigation of IDA on an individual case basis.

Bidirectional endoscopy is also labour intensive, taking up to an hour to complete for each patient, yet over 90% of procedures for IDA will not reveal malignancy. Because it is common, IDA is a major drain on investigational resources, accounting for a substantial proportion of the workload in many endoscopy units—with estimates of up to 20% of all diagnostic examinations [3]. Any manoeuvre to safely reduce the number of necessary investigations has the potential to make a substantial positive impact on both costs and waiting times.

We have previously proposed the IDIOM score as a simple and reliable pre-test predictor of the risk of underlying malignancy that is sufficiently discriminating to be clinically useful for patient-centred counselling [4]. Effective risk stratification is a potentially important clinical tool for two reasons. First, it allows the identification of a very high-risk subgroup who warrant accelerated investigation and can be managed accordingly. Second, it reveals individuals at very low risk who are unlikely to benefit from invasive investigation and may wish to make a considered decision not to proceed. Since there is currently no consensus on the risk threshold warranting investigation for GI cancer in IDA, the IDIOM score is of potential use not just to predict the GI risk and stratify patients in meaningful risk groups, but also to inform the decisions of clinicians and patients when discussing whether invasive investigation is appropriate.

Challenges to the applicability of the IDIOM score to other IDA populations include relatively small proportion of positive cases (8.4% for the Dorset dataset), and differences in predictor definitions, referral pathway and patient characteristics between cohorts in different parts of the country. The external validation exercise reported here was therefore important to confirm that the model underlying the IDIOM score is capable of predicting the risk of underlying GI malignancy in independent external IDA datasets.

Using the combined validation dataset, our results demonstrate that the IDIOM model has good discrimination performance, and of clinical value. The results also suggest that the IDIOM model has no tendency to under- or overestimate risk, and the risk estimates are not systematically too moderate or extreme. Moreover, using the 1.18% cut-off to categorise patients into the ultra-low risk group showed that none of the IDA patients within this group proved to have GI cancer on investigation in any dataset (Dorset, Sheffield and Oxford).

The strength of this study is the inclusion of more than one independent external dataset to validate the model. Also, it represents the first risk prediction model for gastrointestinal cancer in iron deficiency anaemia to be internally and externally validated. Being a retrospective analysis, limitations include our inability to control the size of the study external validation datasets which resulted in a restricted suboptimal evaluation per centre, or to incorporate other variables that might influence GI cancer risk such as family history, previous cancer, race, unintentional weight loss and red meat consumption—though this is the aim of work to develop the model further.

## Conclusion

By analysing two independent datasets, this paper externally validates the IDIOM score risk prediction model, a multivariable logistic regression model developed to predict the risk of gastrointestinal malignancy for patients with iron deficiency anaemia. The assessment of the model performance was evaluated by estimating the measures of discrimination, calibration and net benefit. This external validation exercise has shown promising results regarding using the IDIOM model in predicting the risk of underlying GI malignancy in different IDA populations in the UK; however, further validation of this model in larger datasets would still be useful to confirm the findings from this study.

## Supplementary Information

**Additional file 1: Table S1.** Model coefficients before/after applying different regularisation methods. **Figure S1.** Average 95% confidence interval width for the C-statistic (discrimination) at different effective sample sizes comparing by SD(*LP*) at fixed base probability = 0.084. **Figure S2.** Average 95% confidence interval width for the calibration slope at different effective sample sizes (based on number of events) comparing by SD(*LP*) at fixed base probability =0.084. **Table S2.** Normal ranges for Hb, MCV, ferritin, T.sat in each lab. **Figure S3.** Density plots show the distributions of continuous variables per GI presence/absence in each dataset using IDIOM model. **Figure S4.** Density plots show the distribution of estimated risks per GI presence/absence in each dataset using IDIOM model. **Figure S5.** Risk groups calibration in the combined validation dataset shows the relation between the estimated risks (on the x-axis) and the observed risks (on the y-axis). **Figure S6.** Flexible calibration curve in Oxford dataset shows the relation between the estimated risks (on the x-axis) and the observed proportion of events (on the y-axis). **Figure S7.** Flexible calibration curve in Sheffield dataset shows the relation between the estimated risks (on the x-axis) and the observed proportion of events (on the y-axis). **Figure S8.** Decision curve analysis for GI investigation using Oxford dataset. Grey line: penalised IDIOM model. Black line: “investigate no-one” strategy. Dashed line: “investigate all” strategy. The vertical axis displays standardized net benefit. The horizontal axis shows the risk thresholds. **Figure S9.** Decision curve analysis for GI investigation using Sheffield dataset. Grey line: penalised IDIOM model. Black line: “investigate no-one” strategy. Dashed line: “investigate all” strategy. The vertical axis displays standardized net benefit. The horizontal axis shows the risk thresholds. **Figure S10.** Clinical impact curve for penalised IDIOM risk model using Dorset data, with 95% CIs constructed via bootstrapping. Of 1,000 patients, the heavy black solid line shows the total number who would be deemed low risk for each risk threshold. The blue dashed line shows how many of those would be true negatives. The vertical axis displays standardised net benefit. The two horizontal axes show the correspondence between risk threshold and cost:benefit ratio. **Figure S11.** Clinical impact curve for penalised IDIOM risk model using combined validation data from Oxford and Sheffield, with 95% CIs constructed via bootstrapping. Of 1,000 patients, the heavy black solid line shows the total number who would be deemed low risk for each risk threshold. The blue dashed line shows how many of those would be true negatives. The vertical axis displays standardised net benefit. The two horizontal axes show the correspondence between risk threshold and cost:benefit ratio. **Figure S12.** Clinical impact curve for penalised IDIOM risk model using the Oxford dataset, with 95% CIs constructed via bootstrapping. Of 1,000 patients, the heavy black solid line shows the total number who would be deemed low risk for each risk threshold. The blue dashed line shows how many of those would be true negatives. The vertical axis displays standardised net benefit. The two horizontal axes show the correspondence between risk threshold and cost:benefit ratio. **Figure S13.** Clinical impact curve for penalised IDIOM risk model using the Sheffield dataset, with 95% CIs constructed via bootstrapping. Of 1,000 patients, the heavy black solid line shows the total number who would be deemed low risk for each risk threshold. The blue dashed line shows how many of those would be true negatives. The vertical axis displays standardised net benefit. The two horizontal axes show the correspondence between risk threshold and cost:benefit ratio.

## Data Availability

Further permission and reasonable re-use requests of the data should be made to the authors in each university.

## Abbreviations

AUC: Area under the ROC
CI: Confidence interval
CRC: Colorectal cancer
GI: Gastrointestinal
Hb: Blood haemoglobin concentration
IDA: Iron deficiency anaemia
IDIOM: Iron deficiency as an indicator of malignancy
MCV: Mean cell volume
NB: Net benefit
OGD: Gastroscopy
OR: Odds ratio
ROC: Receiver operating characteristic curve
T.sat: Transferrin saturation
