# Supplementary material for: Broad external validation of a multivariable risk prediction model for gastrointestinal malignancy in iron deficiency anaemia
Source: Diagn Progn Res. 2021 Dec 15;5:23. doi: 10.1186/s41512-021-00112-8 (PMC8672477; doi:10.1186/s41512-021-00112-8)
Supplement: Supplementary file 1 — Additional file 1: Table S1. Model coefficients before/after applying different regularisation methods. Figure S1. Average 95% confidence interval width for the C-statistic (discrimination) at different effective sample sizes comparing by SD(LP) at fixed base probability = 0.084. Figure S2. Average 95% confidence interval width for the calibration slope at different effective sample sizes (based on number of events) comparing by SD(LP) at fixed base probability =0.084. Table S2. Normal ranges for Hb, MCV, ferritin, T.sat in each lab. Figure S3. Density plots show the distributions of continuous variables per GI presence/absence in each dataset using IDIOM model. Figure S4. Density plots show the distribution of estimated risks per GI presence/absence in each dataset using IDIOM model. Figure S5. Risk groups calibration in the combined validation dataset shows the relation between the estimated risks (on the x-axis) and the observed risks (on the y-axis). Figure S6. Flexible calibration curve in Oxford dataset shows the relation between the estimated risks (on the x-axis) and the observed proportion of events (on the y-axis). Figure S7. Flexible calibration curve in Sheffield dataset shows the relation between the estimated risks (on the x-axis) and the observed proportion of events (on the y-axis). Figure S8. Decision curve analysis for GI investigation using Oxford dataset. Grey line: penalised IDIOM model. Black line: “investigate no-one” strategy. Dashed line: “investigate all” strategy. The vertical axis displays standardized net benefit. The horizontal axis shows the risk thresholds. Figure S9. Decision curve analysis for GI investigation using Sheffield dataset. Grey line: penalised IDIOM model. Black line: “investigate no-one” strategy. Dashed line: “investigate all” strategy. The vertical axis displays standardized net benefit. The horizontal axis shows the risk thresholds. Figure S10. Clinical impact curve for penalised IDIOM risk model using Dorset data, w [file 41512_2021_112_MOESM1_ESM.docx]

**Broad External Validation of a Multivariable Risk Prediction Model for GI Malignancy in Iron Deficiency Anaemia**

Orouba Almilaji ^1 2^, Gwilym Webb ^3^, Alec Maynard ^4^, Thomas P Chapman ^5^, Brian SF Shine ^6^, Antony J Ellis ^7^, John Hebden ^8^, Sharon Docherty ^2^, Elizabeth J Williams ^1^, Jonathon Snook ^1^

^1^ Gastroenterology Unit, Poole Hospital, University Hospitals Dorset NHS Foundation Trust, UK

^2^ Department of Medical Science and Public Health, Bournemouth University, UK

^3^ Cambridge Liver Unit, Addenbrooke's Hospital, Cambridge University Hospitals NHS Foundation Trust, UK

^4^ Weston Park Cancer Centre, Sheffield Teaching Hospitals NHS Foundation Trust, UK

^5^ Department of Gastroenterology, Worthing Hospital, University Hospitals Sussex NHS Foundation Trust, UK

^6^ Nuffield Department of Clinical Laboratory Sciences, University of Oxford, UK

^7^ Translational Gastroenterology Unit, NIHR Oxford Biomedical Research Centre, University of Oxford, UK

^8^ Northern General Hospital, Sheffield Teaching Hospitals NHS Foundation Trust, UK

**Corresponding author**: Orouba Almilaji

Medical Science and Public Health Department

Bournemouth University, Bournemouth, UK

E-mail: oalmilaji@bournemouth.ac.uk

ORCID: https://orcid.org/ 0000-0002-0846-5393

**Supplementary Information:**

**Table S1** Model coefficients before/after applying different **regularisation methods**:

|  | **Full model^1^** | **Heuristic^2^** | **Bootstrap^3^** | **Penalised^4^** | **Lasso^5^** |
| --- | --- | --- | --- | --- | --- |
| **Intercept** | -1.84 | -1.80 | -1.85 | -1.77 | -1.84 |
| **Sex (male)** | 0.939 | 0.917 | 0.919 | 0.903 | 0.894 |
| **Age (years)** | 0.057 | 0.056 | 0.056 | 0.055 | 0.049 |
| **MCV (fl)** | -0.033 | -0.032 | -0.032 | -0.032 | -0.030 |
| **Hb (g/l)** | -0.026 | -0.025 | -0.025 | -0.025 | -0.025 |

^1:^ Standard binary logistic model

^2:^ Heuristic shrinkage

^3:^ Bootstrap shrinkage (500 bootstraps)

^4:^ Penalized maximum likelihood using

^5:^ Least absolute shrinkage and selection operator (Lasso)

**Sample size considerations**

Being a retrospective analysis of secondary data meant we did not have any control over the size of the external validation datasets. The number of outcome events in the Oxford and Sheffield datasets were 86, 36 respectively.

Following the simulation-based sample size calculations for external validation of clinical prediction models [1], the anticipated precisions of performance measures were estimated based on the available number of outcome events in the external validation datasets, and on them both combined.

Using a fixed base probability of 0.084 (200/2390 from Dorset dataset), the simulation-based sample size calculations predicted an average 95% CI width for the discrimination value of around 0.16 when the standard deviation of linear predictor SD(LP) is 1 for 36 outcome events (n=429) as can be seen from figure S1. The figure was 0.10 for 86 outcome events (n=1024), and 0.08 for 122 outcome events (n=1452). Accordingly, to achieve a 95% CI width for the C-statistic < 0.1, 86 outcome events should be sufficient to achieve this precise performance measure. This means that the Oxford dataset can be used separately, or it can be combined with the Sheffield dataset to achieve more precise discrimination. A precision of discrimination performance < 0.1 cannot be achieved by using Sheffield dataset alone.

The simulation also showed that for the calibration slope (figure S2), a 95% CI width of 0.8 would be predicted at SD(LP) =1 for 36 outcome events, 0.6 for 86 outcome events, and 0.4 for 122 outcome events. Achieving a 95% CI width for the calibration slope < 0.2, would need about more than 500 outcome events. Thus, to achieve the most possible precise 95% CI width for the calibration slope, the two validation datasets must be combined.

**References:**

1. Snell KIE, Archer L, Ensor J, et al. External validation of clinical prediction models: simulation-based sample size calculations were more reliable than rules-of-thumb. J Clin Epidemiol 2021 Feb 14(135):79-89. doi: 0.1016/j.jclinepi.2021.02.011.

**Figure S1** Average 95% confidence interval width for the C-statistic (discrimination) at different effective sample sizes comparing by SD(LP) at fixed base probability = 0.084.


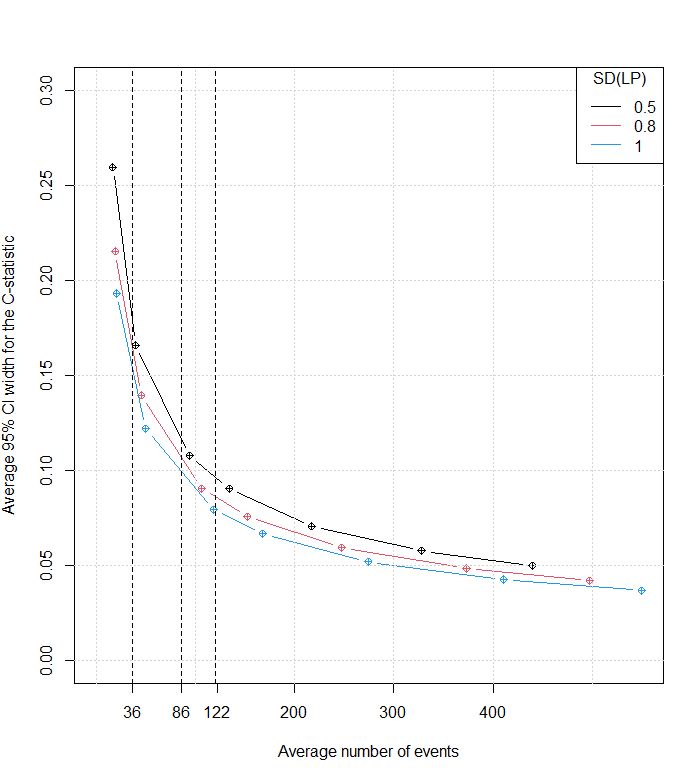


**Figure S2** Average 95% confidence interval width for the calibration slope at different effective sample sizes (based on number of events) comparing by SD(*LP*) at fixed base probability =0.084


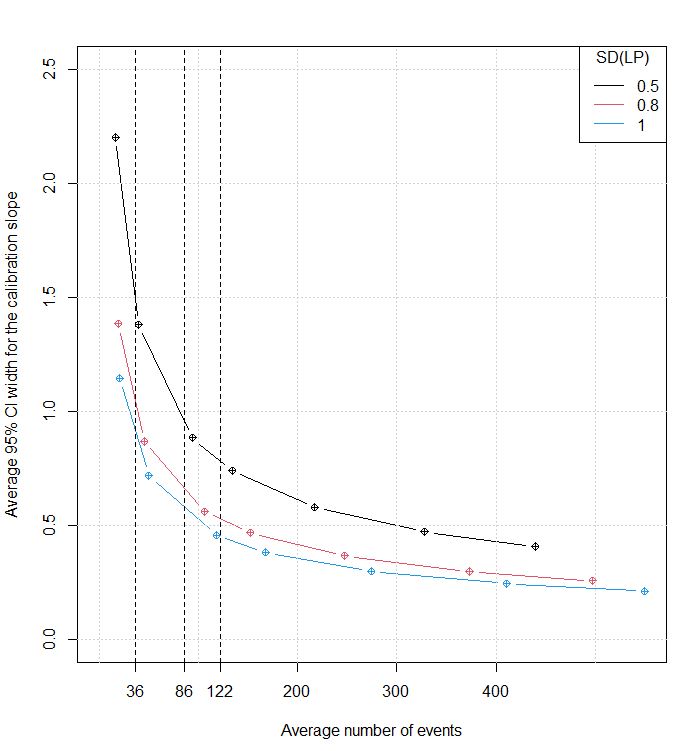


**Table S2** Normal ranges for Hb, MCV, ferritin, T.sat in each lab

|  | **Dorset dataset** | **Oxford dataset** | **Sheffield dataset** |
| --- | --- | --- | --- |
| **Ferritin (ug/l)** | Female: 13-150  Male: 30-400 | Female: 10-200  Male: 20-300 | 31-400 |
| **T.sat (%)** | 15-45 | 16-50 | Female: 15-45  Male: 15-50 |
| **Hb (g/l)** | Female: 115-150  Male: 130-170 | Female: 120-150  Male: 130-170 | Female: 110-147  Male: 131-166 |
| **MCV (fl)** | 78-99 | 83-105 | Female: 80.0-98.1  Male: 81.8-96.3 |

**Figure S3** Density plots show the distributions of continuous variables per GI presence/absence in each dataset using IDIOM model


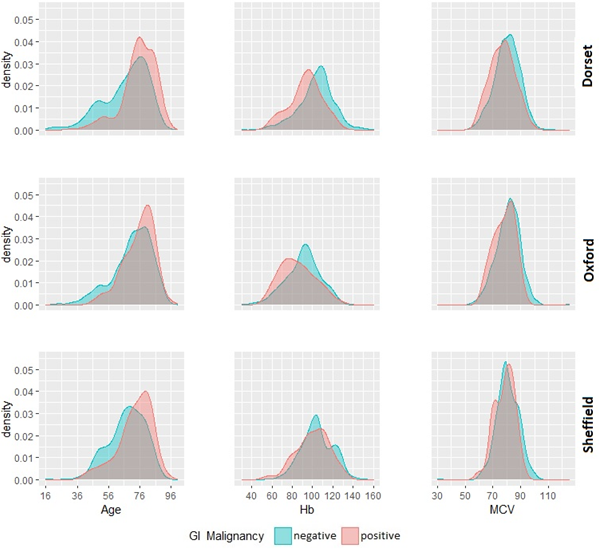


**Figure S4** Density plots show the distribution of estimated risks per GI presence/absence in each dataset using IDIOM model


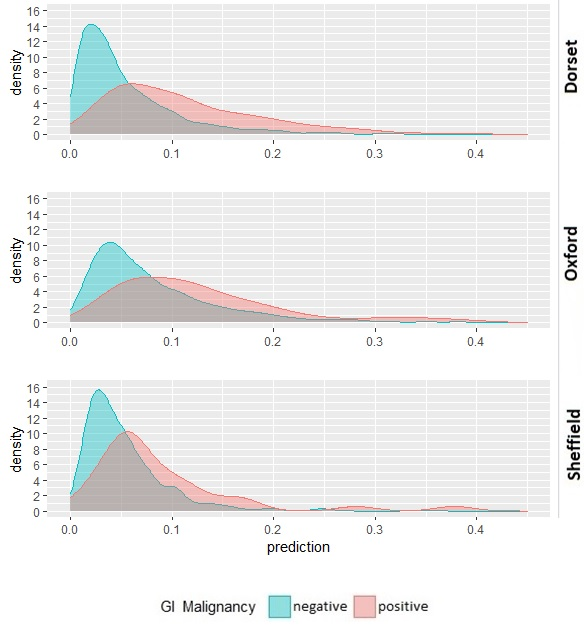


**Figure S5** Risk groups calibration in the combined validation dataset shows the relation between the estimated risks (on the x-axis) and the observed risks (on the y-axis)


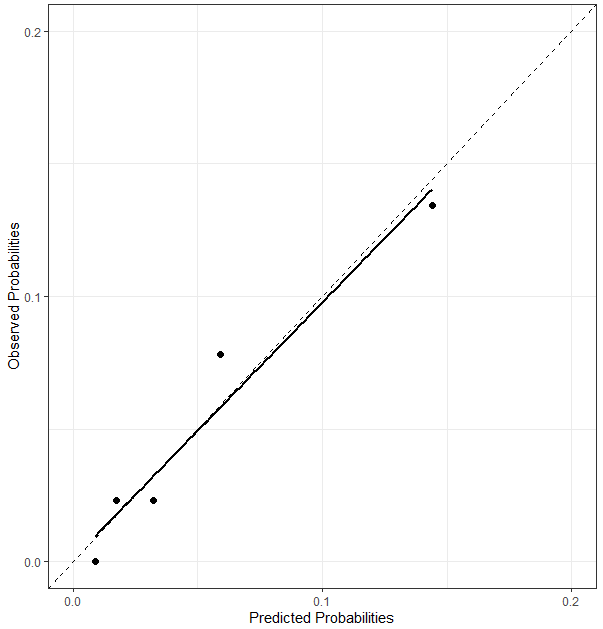


**Figure S6** Flexible calibration curve in **Oxford dataset** shows the relation between the estimated risks (on the x-axis) and the observed proportion of events (on the y-axis)


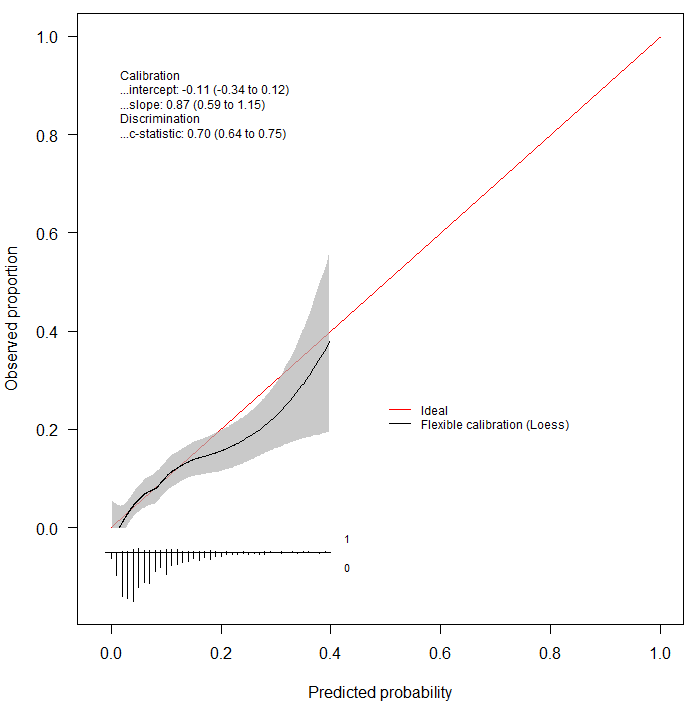


**Figure S7** Flexible calibration curve in **Sheffield dataset** shows the relation between the estimated risks (on the x-axis) and the observed proportion of events (on the y-axis)


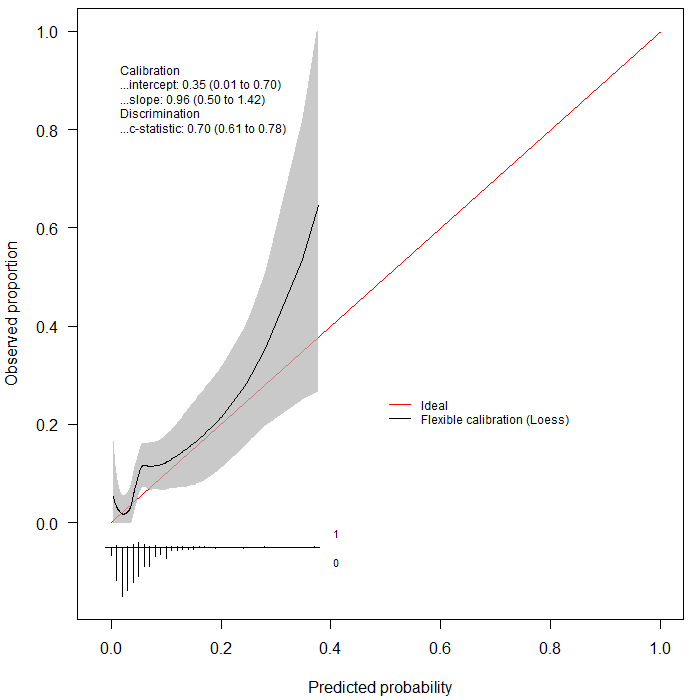


**Figure S8** Decision curve analysis for GI investigation using **Oxford dataset**. Grey line: penalised IDIOM model. Black line: “investigate no-one” strategy. Dashed line: “investigate all” strategy. The vertical axis displays standardized net benefit. The horizontal axis shows the risk thresholds.


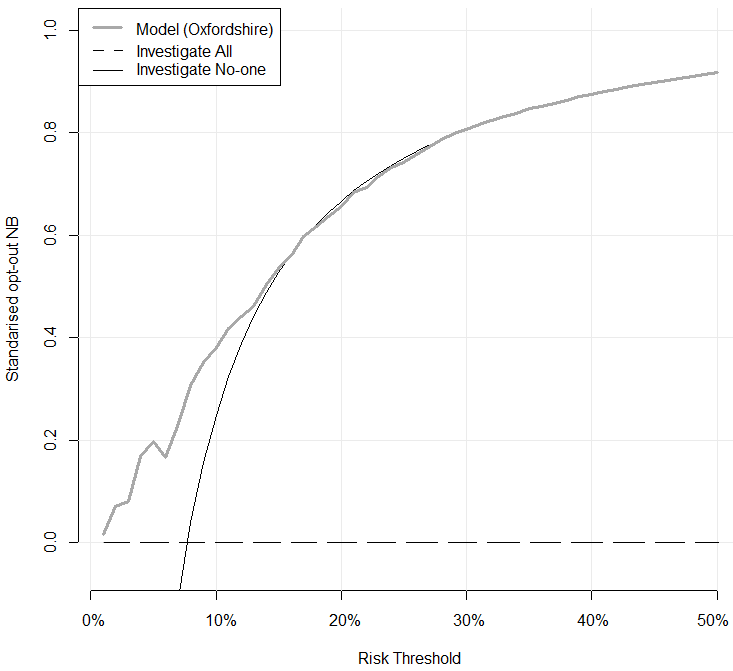


**Figure S9** Decision curve analysis for GI investigation using **Sheffield dataset**. Grey line: penalised IDIOM model. Black line: “investigate no-one” strategy. Dashed line: “investigate all” strategy. The vertical axis displays standardized net benefit. The horizontal axis shows the risk thresholds.


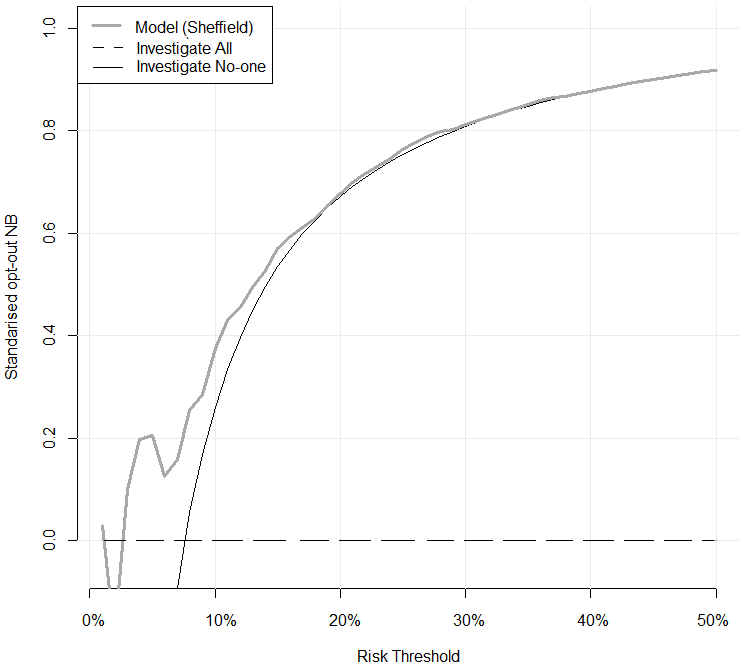


**Figure S****10** Clinical impact curve for penalised IDIOM risk model using **Dorset data**, with 95% CIs constructed via bootstrapping. Of 1,000 patients, the heavy black solid line shows the total number who would be deemed low risk for each risk threshold. The blue dashed line shows how many of those would be true negatives. The vertical axis displays standardised net benefit. The two horizontal axes show the correspondence between risk threshold and cost:benefit ratio.


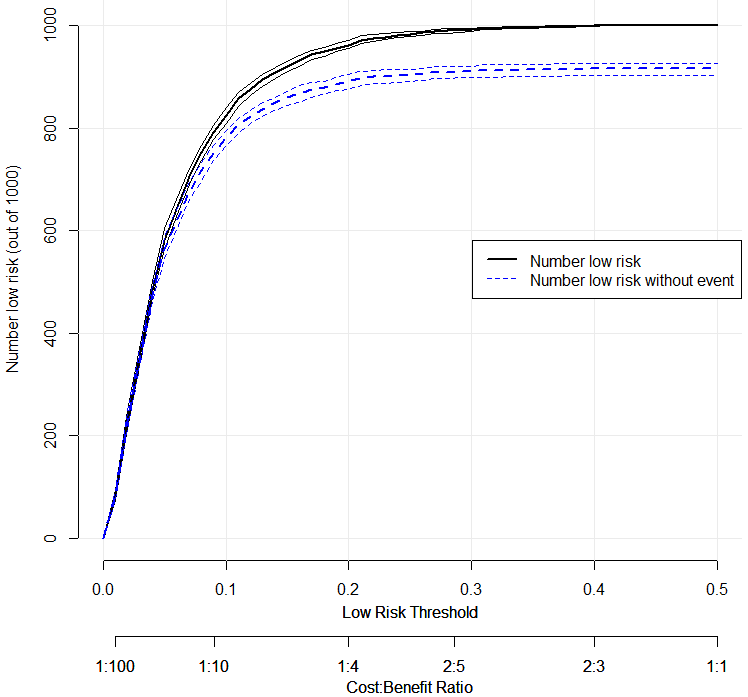


**Figure S11** Clinical impact curve for penalised IDIOM risk model using **combined validation data** from Oxford and Sheffield, with 95% CIs constructed via bootstrapping. Of 1,000 patients, the heavy black solid line shows the total number who would be deemed low risk for each risk threshold. The blue dashed line shows how many of those would be true negatives. The vertical axis displays standardised net benefit. The two horizontal axes show the correspondence between risk threshold and cost:benefit ratio.


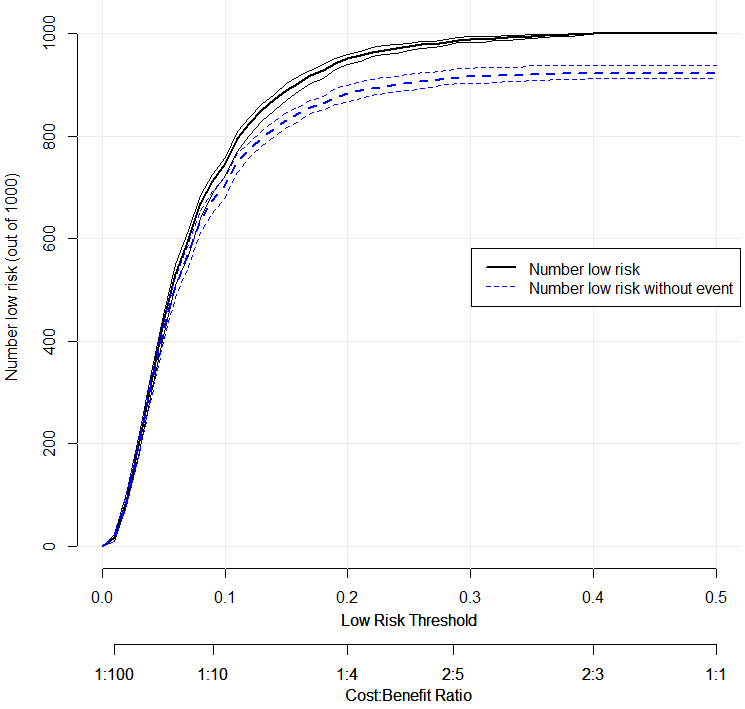


**Figure S12** Clinical impact curve for penalised IDIOM risk model using the **Oxford dataset**, with 95% CIs constructed via bootstrapping. Of 1,000 patients, the heavy black solid line shows the total number who would be deemed low risk for each risk threshold. The blue dashed line shows how many of those would be true negatives. The vertical axis displays standardised net benefit. The two horizontal axes show the correspondence between risk threshold and cost:benefit ratio.

**
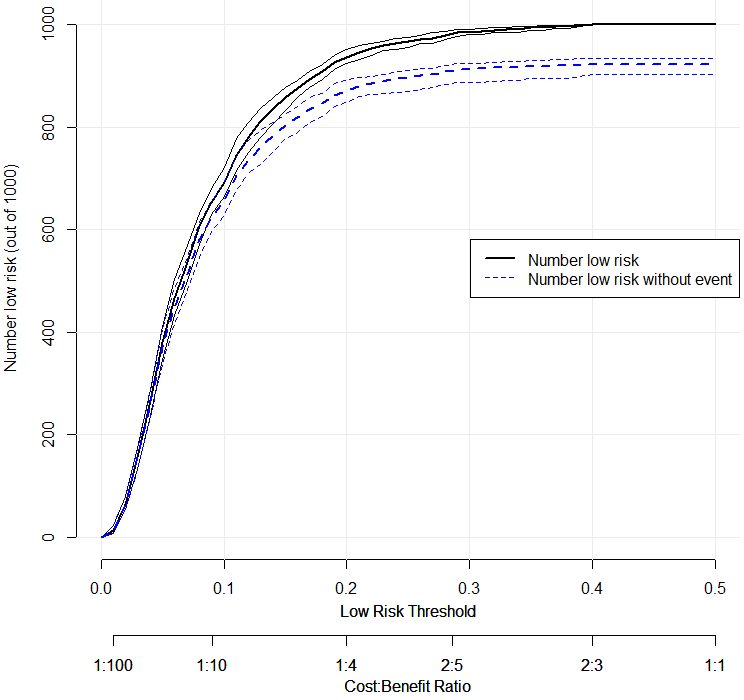
**

**Figure S13** Clinical impact curve for penalised IDIOM risk model using the **Sheffield dataset**, with 95% CIs constructed via bootstrapping. Of 1,000 patients, the heavy black solid line shows the total number who would be deemed low risk for each risk threshold. The blue dashed line shows how many of those would be true negatives. The vertical axis displays standardised net benefit. The two horizontal axes show the correspondence between risk threshold and cost:benefit ratio.

**
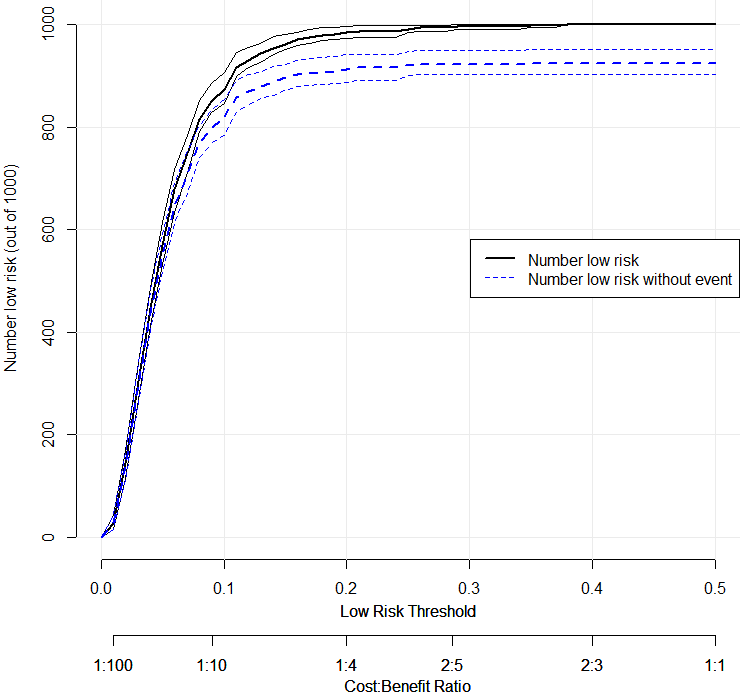
**
